# Genomic Insights of *Alnus*-Infective *Frankia* Strains Reveal Unique Genetic Features and New Evidence on Their Host-Restricted Lifestyle

**DOI:** 10.3390/genes14020530

**Published:** 2023-02-20

**Authors:** Sandra Kim Tiam, Hasna Boubakri, Lorine Bethencourt, Danis Abrouk, Pascale Fournier, Aude Herrera-Belaroussi

**Affiliations:** 1 Université de Lyon, F-69361 Lyon, France, Université Claude Bernard Lyon 1, CNRS, UMR 5557, INRA UMR 1418, Ecologie Microbienne, F-69622 Villeurbanne, France; 2 UMR CNRS 5557 Ecologie Microbienne, INRA UMR 1418, Centre d’Etude des Substances Naturelles, Université Claude Bernard Lyon 1, F-69622 Villeurbanne, France

**Keywords:** *Frankia*, actinorhizal symbiosis, *in planta* sporulation, agmatine deiminase, HUP, gas vesicles, genome reduction

## Abstract

The present study aimed to use comparative genomics to explore the relationships between *Frankia* and actinorhizal plants using a data set made of 33 *Frankia* genomes. The determinants of host specificity were first explored for “*Alnus*-infective strains” (i.e., *Frankia* strains belonging to Cluster Ia). Several genes were specifically found in these strains, including an agmatine deiminase which could possibly be involved in various functions as access to nitrogen sources, nodule organogenesis or plant defense. Within “*Alnus*-infective strains”, Sp+ *Frankia* genomes were compared to Sp− genomes in order to elucidate the narrower host specificity of Sp+ strains (i.e., Sp+ strains being capable of *in planta* sporulation, unlike Sp− strains). A total of 88 protein families were lost in the Sp+ genomes. The lost genes were related to saprophytic life (transcriptional factors, transmembrane and secreted proteins), reinforcing the proposed status of Sp+ as obligatory symbiont. The Sp+ genomes were also characterized by a loss of genetic and functional paralogs, highlighting a reduction in functional redundancy (e.g., *hup* genes) or a possible loss of function related to a saprophytic lifestyle (e.g., genes involved in gas vesicle formation or recycling of nutrients).

## 1. Introduction

Despite its abundance in the atmosphere, nitrogen is the main element limiting plant growth. This is known as the nitrogen paradox. Actually, atmospheric nitrogen (N_2_) is not directly available to plants, as only diazotrophic bacteria are able to fix N_2_ through the action of nitrogenase, a metalloenzyme reducing N_2_ to ammonia (NH_3_). The symbiotic association with such diazotrophic bacteria allows the plant to benefit from an abundant nitrogen source. In return, the plant provides photosynthates to bacteria. This exchange benefits both partners and thus defines the symbiotic relationship between plant and bacteria. This symbiosis between plants and diazotrophic soil bacteria is found in a very limited number of plants and with two types of bacteria: *Rhizobium* and *Frankia*, defining the legume–*Rhizobium* symbiosis and the plant–*Frankia* symbiosis (i.e., actinorhizal symbiosis), respectively.

In both symbiotic models, the microbial symbiotic partner can show a variable degree of host specificity (resulting from multiple interactions involving signaling among bacteria and host plants): some strains establish highly specific interactions with their host, while others are versatile and infect a large spectrum of host plants [1,2]. This host specificity concept has long formed the basis of *Rhizobia* and *Frankia* strain classification into host specificity groups (HSGs, i.e., set of strains nodulating the same compatible host plants). For instance, until the early 1980s, all symbiotic nitrogen-fixing bacteria from leguminous plants were classified in the single genus *Rhizobium*, including six species, *Rhizobium leguminosarum*, *R. meliloti*, *R. trifolii*, *R. phaseoli*, *R. lupini* and *R. japonicum*, matching the cross-inoculation groups [3,4,5,6]. However, many exceptions to this “host specificity rule” have been revealed, and the classification of legume-infective rhizobial strains has undergone great changes based on their characterization by polyphasic taxonomy [5]. On the other hand, host specificity still remains a strong criterion which allows the division of *Frankia* strains into large groups. Strong correlations indeed exist between the taxonomy of *Frankia* strains and their host range [7,8,9]. *Frankia* strains are more precisely classified into four HSGs, three of which contain symbiotic strains. Among them, Cluster I groups *Frankia* strains nodulating plants into three actinorhizal families of the order Fagales: Betulaceae, Casuarinaceae and Myricaceae [7]. This cluster is subdivided into two main subclusters: Ia (often referred to as “*Alnus* strains”) including *Alnus*-infective strains (cultivated or directly identified from *Alnus* nodules) and few strains isolated from *Myrica* or *Comptonia* nodules, and Ic for the narrow host range “*Casuarina* strains” that, under natural conditions, nodulate only *Casuarina* and *Allocasuarina* species in the Casuarinaceae [10]. “*Alnus*-infective strains” from Cluster Ia were long thought to share the same host range (this specificity group concept was confirmed for most *Alnus*-cultured strains, even for strains isolated from *Myrica* and *Comptonia*—Refs. [1,7,11]), until cross-inoculation experiments using crushed nodules as inocula suggested the existence of particular *Alnus*-infective *Frankia* strains with a narrower host range [12,13,14,15,16]). These strains, named “Sp+”, are distinguished from others by their ability to profusely sporulate within the host root nodules (unlike Sp− strains, unable of *in planta* sporulation) [13]. Described in 1978 by Van Dijk [17], they are still culture recalcitrant (none are available in pure culture despite many isolation attempts) [18]. Their narrower host specificity was recently confirmed based on plant-trapping experiments, suggesting a strong host dependence [19]. Recently, Sp+ genomes were obtained directly from *Frankia* spores isolated from nodules of different *Alnus* species and revealed that *Alnus*-infective Sp+ strains represent distinct species within the Cluster Ia, strongly correlated to the *Alnus* species [20,21,22].

The strong influence of host specificity in *Frankia* strain classification and the existence of especial Sp+ *Alnus*-infective strains make the *Frankia* genus, and particularly Cluster Ia, a relevant model to investigate the decisive factors controlling host specificity. To date, little is known about these factors, despite numerous studies. Host specificity in actinorhizal symbioses is in part controlled by the production of an extracellular root hair deforming factor by the bacterial partner. Interestingly, the results obtained by Cérémonie et al. [23] suggest that *Frankia* root hair deforming factor is structurally different from *Rhizobium* nod factors: biochemical bioassays showed that *Frankia* root hair deforming factor is heat-stable, hydrophilic and chitinase resistant. These results were later comforted by the sequencing of *Frankia* genomes, highlighting the absence of *nod* genes similar to the ones found in *Rhizobium* (only some putative nod-like genes were detected in *Frankia* genomes, without any organized clusters) [24,25], except for the genome of *Frankia datiscae* Dg1 from the Cluster II which expressed a nodABC in its host plant [26].

Over the past few years, more than thirty *Frankia* strains covering the diversity of the *Frankia* genus have been sequenced, including uncultured strains, with a large number of them in Cluster I (more than half of published genomes). All these genomes allowed researchers to name at least 15 *Frankia* species, with representatives in each major cluster of the genus, including two Sp+ *Frankia* species in Cluster Ia [22]. Comparative genomic studies have also revealed (i) the metabolic diversity and natural product biosynthesis pathways in *Frankia* strains [27,28,29], (ii) a strong correlation between genome sizes in frankiae and strain saprotrophic capabilities [22,24,30], (iii) the absence of any nitrogen fixation genes within the genome of ineffective *Frankia* strains (i.e., atypical non-nodulating or non-nitrogen-fixing strains) [31,32] and (iv) variable numbers of Horizontal Gene Transfers (HGT) and Insertion Sequence (IS) elements (an indication of the genome plasticity) according to *Frankia* strains [32,33]. However, all these studies generally include no Sp+ genome (whereas a total of 5 Sp+ genomes have been sequenced, [20,22]). In this context, the present study aims to use comparative genomics to investigate Cluster Ia *Alnus*-infective *Frankia* strains, for which several genomes are available, including the only Sp+ genomes described so far, in order to:

(i) identify candidate molecules responsible for host specificity by comparing genomes of Cluster Ia *Frankia* strains to Cluster Ic, Cluster II (the phylogenetically basal cluster of *Frankia*, including strains infective on actinorhizal Cucurbitales, Rosaceae and the Rhamnaceae genus *Ceanothus*), Cluster III (grouping strains nodulate Elaeagnaceae, Rhamnaceae except for *Ceanothus*, and *Gymnostoma* and *Morella*, two outlier genera of the Fagales) and Cluster IV (containing atypical non-nodulating or non-effective strains) [7,8]. We hypothesized that within the shared and specific genes of Cluster Ia strains (i.e., genes shared by all *Frankia* belonging to Cluster Ia and absent in *Frankia* belonging to other clusters Ic, II, III and IV) will be present genes explaining the host specificity.

(ii) investigate for the first time Sp+ *Frankia* genomes in comparison with available Sp− genomes, in order to elucidate original traits, such as their ability to sporulate *in planta* or their non-culturability, and more largely their specific relationships with the host plant. This part of the present work will be reinforced by sequencing a new Sp+ *Frankia* strain, infective on *Alnus cordata* (given that previous sequenced Sp+ strains were infective on *A. glutinosa*, *A. incana* and *A. alnobetula* formerly *A. viridis*) [20,21,22].

## 2. Materials and Methods

### 2.1. Collection of Frankia Genomes from Databases

A total of 32 *Frankia* genomes were collected from databases (Table 1). These genomes included eleven genomes from *Alnus*-infective strains (Cluster Ia), seven genomes from Cluster Ic, four genomes from Cluster II (obligate symbiont, small genome), six genomes from Cluster III and four genomes from Cluster IV (saprophytic strains, including CN3 for the largest genomes). Within Cluster Ia, five *Frankia* genomes belonged to Sp+ (isolated from *A. glutinosa*, *A. viridis* and *A. incana*) and six to Sp− strains.

### 2.2. Genome Sequencing of a New Sp+ Frankia Strain Infective of Alnus cordata

In the present study, we sequenced a new Sp+ genome from nodules collected on a different *Alnus* species: *A. cordata*, endemic to Corsica. Nodules were sampled in November 2011 at the Col de Prato in Corsica (42.426022 latitude, 9.335868 longitude and 920 m elevation) [34]. The *Frankia* genome was sequenced using DNA extracted from a spore suspension isolated from a crushed nodule, as previously described [20]. Genome assembly was realized using Unicycler v0.8.4.0 [35], after reads sorting by nucleotide frequencies to remove potential plant contamination (G+C content ≤ 54%; [20]), and the annotation was conducted on MicroScope platform version 3.10.0 [36]. This new Sp+ *Frankia* genome was named AcoPra (the whole-genome shotgun project has been deposited in DDBJ/EMBL/GenBank under the accession no. PRJEB58754).

Average Nucleotide Identity (ANI) calculations were performed in order to accurately distinguish between strains at the species level in Cluster Ia, using the threshold of 95% for species delineation [37]. The analysis was performed for nine representative *Frankia* genomes of species previously described in Cluster Ia (Table 1), including *Candidatus* Frankia nodulisporulans and *Candidatus* Frankia alpina Sp+ species, using EDGAR 2.0 [38].

### 2.3. Comparative Genome Analyses between Frankia Strains

The identification of homologous protein families between *Frankia* strains was performed with HOGENOM, an automated procedure allowing massive all-against-all similarity searches, gene clustering, multiple alignments computation and phylogenetic trees construction and reconciliation [39]. In the present study, this procedure was used from the nucleic sequences of the 33 *Frankia* genomes to provide high quality homologous families between these genomes. The coding sequences (CDS) were first translated from nucleic genome sequences to generate the corresponding protein sequences. To build families, a similarity search of all proteins against themselves was performed with the BLASTP2 program, the BLOSUM62 amino-acid similarity matrix and a threshold of 10^−4^ for BLAST E-values. The Build_Fam program was used to cluster protein sequences into families. Two protein sequences were included in the same family if remaining HSPs (high-scoring segment = segment with a high level of similarity) covered at least 80% of the protein length and if their similarity was over 50% (two amino-acids are considered similar if their BLOSUM62 similarity score is positive).

COG (Clusters of Orthologous Groups) assignment for each protein was performed using Microscope pipeline from Genoscope (https://mage.genoscope.cns.fr/microscope/home (accessed on 17 November 2022)) and completed through manual annotation using several other softwares. Pfam/InterPro motifs were researched to determine catalytic domains (https://www.ebi.ac.uk/interpro/about/interpro/ (accessed on 17 November 2022)). Signal and transmembrane sequences were identified using signalP6 (https://dtu.biolib.com/SignalP-6 (accessed on 15 December 2022); [40]) and DeepTMHMM (https://dtu.biolib.com/DeepTMHMM (accessed on 15 December 2022), [41]), respectively.

Paralogs were identified using two approaches. The first approach was via KEGG (https://www.genome.jp/kegg/ (accessed on 17 November 2022)) by searching if several enzymes were present in the same metabolic pathways. The second one is based on protein similarity using BlastP in *Frankia alni* ACN14a genome as a query (with full protein length aligned >50% and a % of identity >30%).

## 3. Results and Discussion

### 3.1. Genome Sequencing of a New Alnus cordata-Infective Sp+ Frankia Strain

The final draft assembly for AcoPra consisted of 118 contigs (>500 pb). The maximum length and N50 values of the contigs were 402.97 kb and 142.15 kb, respectively.

Genome completeness was estimated at 98.1%, using CheckM software that assesses the presence of a specific number of markers depending on the studied organism (307 markers for *Frankia* genomes) [42]. The total genome size was 6,392,990 bp, with an overall G + C content of 71.34%. Although this size is slightly larger than that of other *Alnus*-infective Sp+ strains [21,22], it remains among the smallest genomes in the Cluster Ia (generally around 7.5 Mb) and sustains the hypothesis of genome reduction in Sp+ strains.

The AcoPra genome showed median average nucleotide identity (ANI) values higher than 97% with *Frankia nodulisporulans* AgTrS, and equal to or below 78.5% with other *Alnus*-infective *Frankia* species (Table 2). These results suggest that the *A. cordata*-infective Sp+ strain AcoPra from Corsica would belong to *Candidatus* Frankia nodulisporulans sp. nov., previously described as including Sp+ strains infective on *A. glutinosa* from France and Sweden.

The new Sp+ AcoPra genome therefore enriches the genomic data already available for Cluster Ia, including the only Sp+ genomes described so far. We then searched for 33 *Frankia* genomes, among them 12 genomes belonging to Cluster Ia (including the new genome AcoPra), in order (i) to identify candidate molecules responsible for Cluster Ia *Frankia* strain host specificity and (ii) to investigate Sp+ *Frankia* genomes in comparison with Sp− genomes.

### 3.2. Identification of Candidate Molecules Responsible for Host Specificity in Cluster Ia

**Figure 1:**
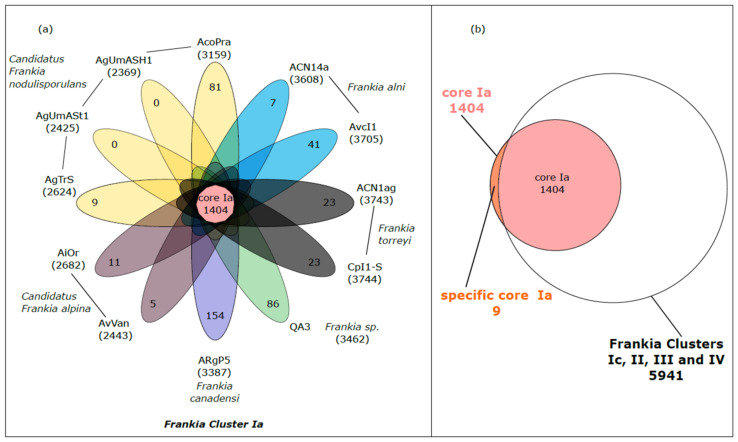
Multigenomic analysis permitted to identify the core genome of *Frankia* belonging to the Cluster Ia and the specific core Ia. (**a**) Flower plot diagram of strains from Cluster Ia. The number within brackets associated to each strain indicates the number of genes in the genome (number of unique CDS). The central circle shows the number of genes common to all strains while the petals show the number of genes specific to each strain. The strains belonging to the same species are shaded with the same color. (**b**) Venn diagram showing the specific core Ia (genes both present in the core-genome of *Frankia* belonging to the Cluster Ia and absent in the pan genome of the *Frankia* belonging to the Clusters Ic, II, III and IV).

In order to identify genes specific to Cluster Ia (*Alnus*-infective), the genomes of the 12 strains belonging to this Cluster Ia were compared to the 21 genomes of strains from Clusters Ic, II, III and IV (Figure 1). The results of the HOGENOM analysis showed the strains belonging to the Cluster Ia had on average 3112 genes (number of unique CDS); this number varied from 2369 for AgUmASH1 to 3744 for CpI1-S. The 12 strains have a conserved core of about 1404 genes (Figure 1a).

Not surprisingly, the number of specific genes (found in only one strain) decreased with the increasing number of representatives within one species. Indeed, *Candidatus* Frankia alpina, *Frankia alni* and *Frankia torreyi* were each represented by two strains and the number of specific genes varied from 5 to 41 (with a mean of 18 specific genes per species); while *Frankia canadensis* and *Frankia* sp. were only represented by one strain and the number of specific genes varied from 86 to 154. Interestingly, the decrease in the number of specific genes with the increase in strains within one species was not observed for *Candidatus* Frankia nodulisporulans. There are four strains belonging to this species (AgUmASH1, AgUmASt1, AgTrs and AcoPra), but the number of specific genes reached 81 for AcoPra.

#### 3.2.1. Specific Core Genome of *Frankia* Belonging to Cluster Ia

Comparing the core genome of *Frankia* belonging to Cluster Ia (pink circle) and the pan genome of *Frankia* belonging to Clusters Ic, II, III and IV, only nine proteins were both present in the core genome of *Frankia* belonging to the Cluster Ia and absent in the pan genome of the *Frankia* belonging to the Clusters Ic, II, III and IV (specific core Ia, orange section) (Figure 1b). Out of these nine proteins, analyses based on sequence similarities allowed us to identify either the structure or the function for six proteins (Table 3).

*Frankia* ACN14a was used as a reference genome since this genome is annotated on KEEG. FRAAL2448 was annotated as a flavodoxin domain-containing protein, FRAAL6541 as a putative signal peptide, FRAAL0164 as an agmatine deiminase, FRAAL0169 as a putative esterase/acetylhydrolase domains-containing protein, FRAAL4245 as a hypothetical integral membrane protein and FRAAL4244 as a sulfite exporter TauE/SafE family protein. FRAAL4245 and FRAAL4244 are located one next to the other in the *Frankia* genome. Protein structure prediction (i.e., DeepTMHMM) identified both proteins as transmembrane proteins; moreover, FRAAL4244 was proposed as a sulfite exporter involved in taurine metabolism (TauE/SafE). As reviewed by Mosier et al. [43], taurine is involved in numerous physiological functions across various lineages; it is a particularly effective osmoregulator and is used as a compatible solute by a variety of microorganisms; moreover, some microbes can use taurine as a source of carbon, nitrogen and sulfur. The use of taurine as a nutrient source was highlighted in *Actinobacteria* in a recent study where the growth of *Marmoricola* sp. TYQ2 (a deep-sea actinobacteria) was significantly promoted by the supplement of taurine [44].

FRAAL0164 and FRAAL0169 are two other genes located close to each other on the *Frankia* genome, indicating that they could be involved in the same metabolic function. While little can be said about FRAAL0169 (i.e., annotated as a putative esterase/acetylhydrolase domains-containing protein), FRAAL0164 caught our attention since it was annotated as an agmatine deiminase and, consequently, due to its potential action in the degradation of agmatine.

#### 3.2.2. Agmatine Deiminase

Among the nine genes found in *Frankia* belonging to Cluster Ia and absent in the pan genome of the *Frankia* belonging to Clusters Ic, II, III and IV, the FRAAL0164 was annotated as an agmatine deiminase (AgD). The lowest percentage of similarity (Clustal Omega alignment tool; [45]) for AgD was observed when comparing ARgP5 and the strains belonging to the species *Candidatus* Frankia nodulisporulans (77.1–77.2% similarity) while the percentage of similarity was on average 85.35% for the 12 strains from Cluster Ia (Table 4).

These results show that AgD is a high conserved protein within *Frankia* strains belonging to Cluster Ia. High conserved proteins carry a very important function, which we hypothesized was our case.

**Figure 2:**
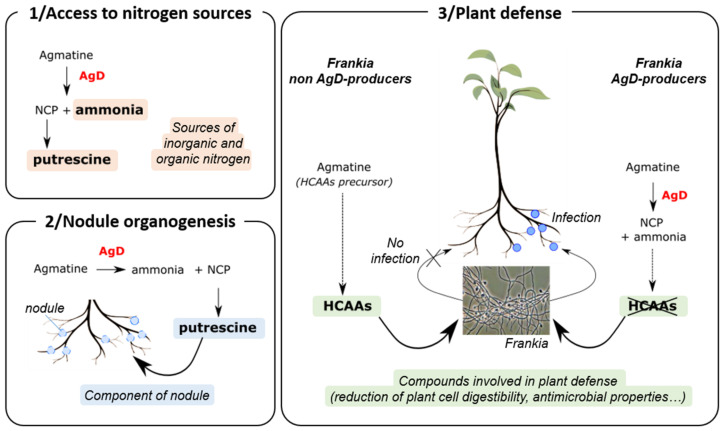
Proposed roles for agmatine deiminase (AgD) in the relationship *Frankia*/*Alnus* regarding: 1. Access to nitrogen: AgD produced by *Frankia* could be used in order to degrade agmatine found in the plant. The enzyme could thus allow *Frankia* to access putrescine (via the conversion of NCP into putrescine) and ammonia as sources of nitrogen, 2. Nodule organogenesis: putrescine obtained from NCP after degradation of agmatine by AgD could be used in nodule development, as putrescine is one of the main polyamine in roots and nodules of actinorhizal and 3. Plant defense: Hydroxycinnamic acid amides (HCAAs) produced by *Alnus* from agmatine are secondary metabolites involved in the defense of plants against pathogens. Production of AgD by *Frankia* AgD producers leads to the degradation of agmatine into N-carbomoyl-putrescine and ammonium and puts a stop to the production of HCAAs. The absence of HCAAs makes possible the infection by *Frankia* and the subsequent formation of nodules.

The AgDs catalyze the deimination of agmatine (i.e., decarboxylated arginine) to form N-carbamoyl putrescine (NCP) and ammonia [46]. We can hypothesize that the AgD produced by *Frankia* could be used in order to degrade agmatine found in the plant (Figure 2). The enzyme could thus allow *Frankia* to produce putrescine (via the conversion of NCP into putrescine) and use ammonia as sources of nitrogen. Actually, studies have shown that *Frankia* strains can use a variety of organic and inorganic sources of nitrogen for growth [10], including putrescine [47]. Moreover, putrescine was identified as one of the three main polyamine (together with spermidine and spermine) in roots and nodules of legumes and of actinorhizals [48,49,50], suggesting an association between polyamines and nodule development [47].

We could also hypothesize that AgD plays a crucial role in *Frankia* infection to circumvent plant defense. Actually, agmatine is a precursor of several secondary metabolites, such as hydroxycinnamic acid amides (HCAAs) produced by plants [51]. HCAAs are a widely distributed group of plant secondary metabolites with a role in several growth and developmental processes (including floral induction, flower formation, sexual differentiation, tuberization, cell division and cytomorphogenesis); they are also involved in plant defense against pathogens [52]. The HCAAs structure is characterized by the association of at least one hydroxycinnamic acid derivative (e.g., p-Coumaroyl-CoA, caffeoyl-CoA, Feruloy-CoA…), which is linked through an amide bond to an aromatic monoamine (e.g., tyramine, dopamine, serotonin…) or an aliphatic polyamine (e.g., agmatine, putrescine, spermidine…) [53]. The combination of different hydroxycinnamic acid and amine moieties together with the possibility of one to four N-substitutions on aliphatic polyamines are responsible for the broad structural diversity in phenolamides. Muroi et al. [51] have shown that mutants of *Arabidopsis thaliana* that do not accumulate HCAAs derived from agmatine and putrescine (p-Coumaroylagmatine, Feruloylagmatine, p-Coumaroylputrescine and Feruloylputrescine) were much more sensitive to *Altenaria brassicicola* infection compared to wild-type, suggesting that these four HCAAs play a crucial role in the infection process.

Regarding their roles as secondary metabolites involved in plant defense against pathogens, we hypothesized that HCAAs derived from agmatine and putrescine potentially produced by *Alnus* prevent infection by *Frankia* non-AgD producers, as illustrated in Figure 2.

On the contrary, *Frankia* AgD producers would have the capability to degrade agmatine into NCP and ammonia; this degradation would prevent the production of HCAAs or strongly reduce HCAAS biosynthesis. In both cases, the decrease in HCAAs production allows the infection by *Frankia* and the subsequent formation of root nodules (Figure 2b).

HCAAs are involved in plant defense by reducing plant cell digestibility by deposition in cell walls [52] and/or by having antimicrobial effects such as the suppression of or reduction in hyphal elongation [51,54,55,56]. We hypothesize the HCAAs produced by *Alnus* will have similar effects on *Frankia* (reduction in the elongation of hyphae), hence preventing *Frankia* infection.

In conclusion, nine genes were specifically found in *Frankia* from Cluster Ia. Among them, FRAAL0164 was annotated as an AgD. This enzyme could play a central role in the *Frankia*/*Alnus* relationship by degrading agmatine into NCP and ammonia. These roles could concern: 1. access to nitrogen sources by providing putrescine (via NCP) and ammonia to *Frankia* and/or 2. nodule organogenesis by using putrescine (i.e., one on the main polyamines in roots and nodules of legumes and of actinorhizals), as well as 3. plant defense by stopping the production of HCAAs derived from agmatine and putrescine.

### 3.3. What Genome Comparison Tells Us about Sp+ Alnus-Infective Frankia Strains

In addition to identifying candidate molecules responsible for Cluster Ia *Frankia* strain host specificity, the second objective was to investigate Sp+ *Frankia* genomes in comparison with Sp− genomes. The narrower host specificity observed in Sp+ strains [19], combined with the fact that they have never been cultured despite numerous attempts, suggests they could be dependent on the host plant for a large part of their life cycle. Several hypotheses have been proposed regarding the *in planta* sporulation strategy of Sp+ *Frankia* strains, among them a possible evolution of Sp+ *Frankia* strains towards an obligatory symbiont status [12,21,22,57]. Under this hypothesis, the early abundant production of spores into host plant cells could allow a massive spore release into the soil during nodule decay and promote the subsequent root vicinity invasion. Indeed, the sporulation *in planta* would enable Sp+ strains to survive and disseminate outside the host, and to infect new roots without the need for saprophytic growth.

A substantial genomic purge of Sp+ strains in Cluster Ia was previously reported, supporting the obligate symbiont scenario previously discussed [21,22]. In the present study, we sequenced a new Sp+ *Frankia* genome from Cluster Ia. Although its size was slightly larger than that of other *Alnus*-infective Sp+ strains [21,22], it remains among the smallest genomes in Cluster Ia (generally around 7.5 Mb), sustaining the hypothesis of a genome reduction in Sp+ strains in two independent lineages. At this stage in the work, it remains crucial to elucidate lost genomic regions in Sp+ strains. Analyzing lost genes could, for instance, comfort the hypothesis that Sp+ strains would have evolved into obligate symbionts.

In the present study, a comparison between Sp+ and Sp− genomes from Cluster Ia *Alnus*-infective *Frankia* strains was performed with HOGENOM. This analysis allowed us to identify 88 protein sequence families found especially in the six Sp− genomes without orthologs in the six Sp+ genomes (Table 5) (it should be noted that the analysis did not reveal any sequence family present in Sp+ genomes without orthologs in Sp− genomes).

These 88 sequence families were characterized based on their COG affiliation or their cellular localization (Table 5). This analysis revealed four major pieces of information that could support the hypothesis of Sp+ strain evolution towards an obligatory symbiont status:

#### 3.3.1. The Loss of Transcription-Associated Protein Sequences in Sp+ *Frankia* Genomes

Based on COG affiliation, we observed 15.9% of lost protein sequences in Sp+ genomes (14 out of 88 sequences) were associated with the “Transcription” category (COG K) (Table 5). For example, several genes encoding transcriptional regulators, including LuxR (e.g., FRAAL4738), MarR (e.g., FRAAL3611), TetR (e.g., FRAAL3977) or putative two-component system response regulators (e.g., FRAAL1658) were observed only in Sp− genomes. Such a purge in genes encoding transcriptional factors and particularly activators has already been reported in the genome reduction bacteria. This phenomenon was hypothesized to reflect a host-restricted lifestyle that requires the symbiont to less finely regulate its gene expression to respond and adapt to changing environmental conditions (e.g., biotic and abiotic stresses) [58,59]. In the case of Sp+ strains, a reduction in the number of transcription-associated sequences could therefore indicate a narrower interaction with the host plant compared to Sp− strains, comforting the hypothesis of the evolution towards an obligate symbiotic status.

#### 3.3.2. A Reduced Secretome in Sp+ *Frankia* Strains

The 88 protein families without orthologs in Sp+ genomes were analyzed regarding their localization in the cell (Table 5). Twenty-six percent of these families were predicted as transmembrane proteins or secreted proteins (indicated in Table 5 as “TM” and “SP”, respectively), including various receptors, transporters and secreted enzymes. For example, orthologs to FRAAL3906 and FRAAL3907 organized in a synton (encoding transporters) were observed only in Sp− genomes. In other words, a significant part of lost sequences in Sp+ genomes would be related to the secretome. A previous comparison of predicted secretomes between plant symbiotic bacteria, in this case *Frankia* strains, and soil bacteria reported a secretome size reduction in the symbiotic bacteria [60]. This reduction was discussed as a consequence of the bacterial adaptation to plant endosymbiotic lifestyle that may require fewer secreted proteins [60,61]. Such a reduction in Sp+ *Frankia* genomes could therefore be additional evidence in favor of the hypothesis of their obligate status.

#### 3.3.3. The Potential Loss of Saprophytic Functions in Sp+ *Frankia* Strains

Interestingly, several protein encoding sequences lost in Sp+ genomes did not present paralogs in Sp− genomes. The loss of these sequences could lead to the loss of functions in Sp+ *Frankia* strains, in absence of divergent genes ensuring the same function.

Genes encoding putative gas vesicles illustrate this situation. Gas vesicles are intracellular air-filled organelles of around two nanometers, composed solely of proteins to trap gas to provide buoyancy to cells in a watery environment. Our analysis revealed that protein encoding sequences involved in the formation of gas vesicle formation (FRAAL3025 annotated “*gvpA*” and FRAAL3026 annotated “*gvpF*”) were absent in Sp+ genomes compared to Sp− genomes. Their absence was recently reported based on the first three sequenced Sp+ genomes [22], and it is supported in the present study including double Sp+ genomes. Gas vesicle proteins could possibly be used for floatation of free-living bacteria on the soil watertable. We hypothesize that obligate plant endosymbionts would not require such a function, and thus the absence of *gvp* genes in Sp+ strains could evidence their high dependance on the host.

In addition to gas vesicle formation, another striking example of encoding protein sequences present in single copies in Sp− genomes, which was lost in Sp+ genomes is FRAAL3502 putatively encoding a 3-ketosteroid 9alpha-monooxygenase. This gene is involved in the cholesterol degradation pathway [62,63]. This pathway could allow Sp− *Frankia* strains to metabolize cholesterol as a carbon and energy source, and it could be involved in strain ability to scavenge nutrients in soil. Steroid degradation is indeed a critical process for biomass decomposition in soil and plant rhizosphere, and it has been found mostly due to actinobacteria, to which the genus *Frankia* belongs [64]. The loss of this gene in Sp+ genomes could suggest a loss of saprophytic abilities in Sp+ strains: Sp+ strains would have lost the ability to metabolize cholesterol in soil but, as obligate symbionts, they would still require host cholesterol for intracellular survival (as previously reported for *Mycobacterium leprae*) [65].

More anecdotally, other sequences encoding proteins with potential functions in the use of soil nutrient and energy resources were also missing in Sp+ genomes, with among them one sequence coding acid phosphatase (SurE, FRAAL0277), considered a predominant form of extracellular phosphatases in soils [4,66].

#### 3.3.4. The Loss of Genetic and Functional Redundancy in Sp+ Genomes

Interestingly, 27% of genes lost in Sp+ genomes present paralogs in Sp− genomes, suggesting a functional redundancy. We can hypothesize that their absence in Sp+ genomes could have few or no effects on their phenotype.

The *hup* genes are the striking example of the presence of paralogs in *Frankia* genomes, some of which have been lost in Sp+ strains. The *hupS* and *hupL* genes encode the hydrogenase structural subunits. With the other *hupABCDEF* genes encoding enzymes involved in the recruitment and incorporation of metallic groups, they form the hup gene cluster. Uptake hydrogenases catalyze the oxidation of hydrogen to protons and electrons in order to supply them to the respiratory chain to produce energy. In diazotrophic bacteria, the nitrogen-fixing activity produces hydrogen that can be consumed to yield energy for other metabolic pathways in the cell [67]. Two sets of uptake hydrogenase genes, organized in synton #1 and synton #2, have been described in *Frankia* [68,69]. The uptake hydrogenase synton #1 was described as more expressed under free-living conditions, whereas hydrogenase synton #2 was mainly involved in symbiotic interactions [68]. In our analysis, *hupDSL* genes belonging to the synton #1 were not present in Sp+ genomes. This could suggest that synton #1 would be no longer needed or useful for the Sp+ strain lifestyle, converging with the hypothesis of their obligate status. Under this hypothesis, they would have lost synton #1, but still require synton #2 to take up hydrogen inside host cells.

In addition to hydrogenase function improving nitrogen fixation, we found gene redundancy assigned to functions involved in the metabolization of different sources that could be associated to life in cell free conditions. Several genes belonging to the “Energy production and conversion” COG were, for example, recovered from the list of lost genes in Sp+ genomes, such as FRAAL3448 or FRAAL4787 encoding a putative Glycerophosphoryl diester phosphodiesterase (indicated as “GlpQ” in Table 5) and putative N-glycosyltransferase, respectively. GlpQ is a protein able to hydrolyze glycerophosphodiester bonds [70] of phospholipid fatty acids, composing cell membranes in all organisms other than archaea, in order to access carbon and phosphate sources [71]. In parallel, the glycosyltransferases classified as GT1 according to the Cazy database (http://www.cazy.org/ (accessed on 17 November 2022) [72]) catalyze the transfer of a sugar moiety from an activated donor sugar onto acceptor molecules such as glycolipids, flavonoids or macrolides [73]. The important role of this enzyme is to resist toxic products produced by bacteria in the environment [74,75,76]. Thus, those enzymes could participate in the bacterial homeostasis to reduce biotic stress or to access new nutrients.

## 4. Conclusions

The present study aimed to use comparative genomics to explore the host specificity of both “*Alnus*-infective strains” and Sp+ *Frankia*. Several genes were specifically found in “*Alnus*-infective strains”, including an agmatine deiminase which could possibly be involved in various functions such as access to nitrogen sources, nodule organogenesis or plant defense. In order to test these functions, the heterologous expression of AgD could be used in future studies to produce this agmatine deiminase to confirm its biochemical function. Its deletion in the *Frankia* genome is a striking demonstration of this, provided that the technique is developed in this model, which is not yet the case.

A total of 88 protein families were lost in the Sp+ genomes. This loss included (i) transcriptional factors, (ii) transmembrane and secreted proteins, (ii) genetic and functional paralogs highlighting a reduction in functional redundancy (genes that copy number decreased, e.g., *hup* genes) and (iv) a possible loss of function (genes with loss of all copies, e.g., genes involved in gas vesicle formation or recycling of nutrients). It highlights a purge of genes related to saprophytic life and comforts the hypothetical status of obligatory symbiont of Sp+ strains. At this stage in the work, it could be interesting to test if lost genes could indeed play a role in *Frankia* saprophytic life. The comparison of their expression when *Frankia* is free-living in soil (e.g., in inoculated soil with *Frankia* Sp− strains) versus under a symbiotic state (e.g., in Sp− nodules) through transcriptomic-based analyses (e.g., qPCR or RNAseq analysis) could, for example, be tested.

To date, we still do not know what explains the ability of Sp+ strains to sporulate *in planta*. Our comparative genomic analysis did not provide new clues to this question (no protein sequence family specific to Sp+ genomes (i.e., without orthologs in Sp− genomes) was revealed). Remember, however, that based on Sp− *Frankia* strains’ ability to sporulate in vitro, it was hypothesized that both Sp+ and Sp− strains have sporulation-associated genes in their genomes, but molecular factors (e.g., transcriptional factors) could suppress the sporulation capacity of Sp− *Frankia* strains *in planta* and allow in Sp+ strains the expression of sporulation inside nodules [77]. To elucidate the question of *in planta* sporulation ability, it would therefore be more worthwhile to follow the expression of *Frankia* genes identified as involved in sporulation in Sp+ versus Sp− nodules [77].

## Figures and Tables

**Table 1:** List of the 32 *Frankia* genomes collected.

*Frankia* Strain	Cluster	Genome Size (pb)	Number of Contig	CheckM Completeness (%)	GC%	Total Number of CDS *	Accession Number
*Candidatus* Frankia nodulisporulans AgTrS	Ia	4,943,752	612	98.09	71.61	5178	NZ_CADCWS010000612.1
*Candidatus* Frankia nodulisporulans AgUmASt1	Ia	4,311,763	304	98.63	71.34	3665	CADDZU010000001
*Candidatus* Frankia nodulisporulans AgUmASH1	Ia	4,285,763	231	97.54	71.23	3652	CADDZW010000001
*Candidatus* Frankia alpina AiOr	Ia	5,571,616	669	99.38	71.57	6192	GCA_902806485
*Candidatus* Frankia alpina AvVan	Ia	5,009,155	1233	98.10	71.34	5157	GCA_004803575
*Frankia alni* ACN14a	Ia	7,497,934	1	100	72.83	6714	NC_008278.1
*Frankia alni* AvcI1	Ia	7,741,902	77	99.65	72.60	7255	LJFZ01000001.1
*Frankia* sp. QA3	Ia	7,590,853	120	100	72.59	7307	CM001489.1
*Frankia torreyi* CpI1-S	Ia	7,639,958	153	99.38	72.43	7201	JYFN00000000.1
*Frankia torreyi* ACN1ag	Ia	7,521,047	108	99.37	72,50	5687	LJPA01000001.1
*Frankia canadensi* ARgP5	Ia	7,730,285	568	99.73	72.39	7500	OESX01000001
*Frankia casuarinae* CcI3	Ic	5,433,628	1	99.59	70.08	5593	CP000249.1
*Frankia casuarinae* CcI6	Ic	5,592,323	138	99.59	69.99	5837	GCA_000503735.2
*Frankia casuarinae* Thr	Ic	5,298,125	184	_	_	4654	NZ_JENI00000000.1
*Frankia casuarinae* Allo2	Ic	5,352,110	110	_	70.00	4368	GCA_000733325.1
*Frankia casuarinae* BR	Ic	5,227,240	180	_	_	6478	NZ_LRTJ00000000.1
*Frankia casuarinae* CeD	Ic	5,004,600	120	_	70.10	3937	GCA_000732115.1
*Frankia casuarinae* KB5	Ic	5,455,564	420	97.40	70.10	4915	NZ_MRUJ00000000.1
*Candidatus* Frankia datiscae Dg1	II	5,341,139	1	98.36	70.04	5472	CP002801
*Frankia coriaria* BMG5.1	II	5,806,763	116	95.31	70.24	6487	JWIO00000000
*Candidatus* Frankia californiensis Dg2	II	6,180,138	2742	89.39	67.99	7838	FLUV00000000
*Frankia meridionalis* Cppng1	II	4,858,260	1	_	68,10	4968	PRJEB19438
*Frankia discariae* BCU110501	III	7,907,741	200	100	72.39	7567	ARDT00000000
*Frankia* sp. EAN1pec	III	8,982,042	1	100	71.15	9063	NC_009921.1
*Frankia* sp. EUN1f	III	9,392,240	396	98.37	70.81	9728	ADGX01000001.1
*Frankia elaeagni* BMG5.12	III	7,602,436	136	98.62	71.67	6977	ARFH00000000
*Frankia irregularis* G2	III	9,538,404	83	99.46	70.95	8663	FAOZ00000000
*Frankia soli* NRRL B-16219	III	8,032,739	289	_	71.70	7114	MN238860.1
*Frankia inefficax* EuI1c	IV	8,815,781	1	100	72.31	8099	CP002299.1
*Frankia* sp. DC12	IV	6,884,336	12	100	71.93	6630	KQ031391.1
*Frankia saprophytica* CN3	IV	9,978,692	2	98.35	71.81	9262	AGJN00000000
*Frankia asymbiotica* M16386	IV	9,453,064	174	100	71.97	8884	MOMC00000000

* CDS = Coding sequences.

**Table 2:** Median ANI values between AcoPra and other *Alnus*-infective *Frankia* species in Cluster Ia.

	*Frankia* sp. QA3	*Candidatus* Frankia Alpina AiOr	*Candidatus* Frankia Alpina AvVan	*Frankia alni* ACN14a	*Frankia alni* AvcI1	*Frankia torreyi* ACN1ag	*Frankia torreyi* CpI1	*Frankia canadensis* ARgP5	*Frankia* sp. AcoPra	*Candidatus* Frankia Nodulisporulans AgTrs
***Frankia* sp. QA3**	100.0	90.5	91.1	89.9	89.8	90.8	90.7	79.9	78.0	78.9
***Candidatus* Frankia alpina AiOr**	90.1	100.0	99.3	88.3	88.3	89.3	89.2	79.6	77.7	78.8
***Candidatus* Frankia alpina AvVan**	90.6	99.3	100.0	88.8	88.8	89.6	89.6	80.5	78.7	79.4
***Frankia alni* ACN14a**	90.0	88.9	89.5	100.0	99.7	92.1	92.1	79.6	77.8	78.9
***Frankia alni* AvcI1**	89.9	88.8	89.4	99.7	100.0	92.0	92.0	79.4	77.7	78.9
***Frankia torreyi* ACN1ag**	90.8	89.8	90.3	92.1	92.0	100.0	99.9	79.5	77.7	78.8
***Frankia torreyi* CpI1**	90.9	89.8	90.3	92.2	92.0	99.9	100.0	79.4	77.7	78.8
***Frankia canadensis* ARgP5**	79.9	80.4	81.3	79.3	79.4	79.6	79.4	100.0	78.5	79.4
***Frankia* sp. AcoPra**	77.7	78.0	78.9	77.5	77.3	77.3	77.3	78.3	100.0	**98.0**
***Candidatus* Frankia nodulisporulans AgTrs**	78.0	78.6	79.3	78.0	77.8	77.8	77.7	78.5	**97.9**	100.0

**Table 3:** Genes both present in the core genome of *Frankia* belonging to the Cluster Ia and absent in the pan genome of the *Frankia* belonging to the Clusters Ic, II, III and IV (specific core Ia). Label, Begin, End and Length are given for *Frankia* ACN14a as a reference genome. “SP” and “TM” for Secreted Proteins and Transmembrane Proteins, respectively.

Product	Localization	EC Number	Pathway	*Frankia alni* ACN14a			
				N° Accession	Begin	End	Length (pb)
Flavodoxin domain-containing protein				FRAAL2448	2,667,169	2,667,918	750
Putative signal peptide	SP			FRAAL6541	7,118,052	7,118,477	426
Hypothetical protein				FRAAL4761	5,156,216	5,157,130	915
Hypothetical protein	SP			FRAAL1649	1,769,411	1,769,734	324
Hypothetical protein				FRAAL0667	728,649	729,065	417
Agmatine deiminase		EC:3.5.3.12	arginine catabolism	FRAAL0164	158,747	159,802	1056
Putative esterase/acetylhydrolase domains-containing protein	SP			FRAAL0169	163,780	164,418	639
Hypothetical integral membrane protein	TM			FRAAL4245	4,608,083	4,608,523	441
Sulfite exporter TauE/SafE family protein	TM			FRAAL4244	4,607,181	4,608,086	906

**Table 4:** Percent Identity Matrix calculated from AgD protein sequences of the 12 *Frankia* strains from Cluster Ia using the Clustal Omega alignment tool.

Species	Strain	AcoPra	AgTrS	AgUmASt1	AgUmASH1	ACN14a	AvcI1	CpI1-S	ACN1ag	AvVan	AiOr	ARgP5	QA3
***Candidatus* Frankia nodulisporulans**	**AcoPra**												
	**AgTrS**	98.82											
	**AgUmASt1**	98.82	100										
	**AgUmASH1**	98.82	100	100									
***Frankia alni***	**ACN14a**	79.01	79.17	79.17	79.17								
	**AvcI1**	78.4	78.57	78.57	78.57	99.43							
***Frankia torreyi***	**CpI1-S**	79.32	79.46	79.46	79.46	93.16	92.59						
	**ACN1ag**	79.63	79.76	79.76	79.76	93.45	92.88	99.72					
***Candidatus* Frankia alpina**	**AvVan**	79.32	79.46	79.46	79.46	90.88	90.31	90.03	90.31				
	**AiOr**	79.94	80.06	80.06	80.06	90.88	90.31	90.03	90.31	98.86			
***Frankia canadensi***	**ARgP5**	77.23	77.15	77.15	77.15	80.12	79.54	79.83	80.12	78.1	78.1		
***Frankia* sp.**	**QA3**	80.56	80.36	80.36	80.36	91.45	90.88	90.88	91.17	93.45	93.45	80.98	

**Table 5:** List of specific genes found in Sp− genomes and absent in Sp+ genome from Cluster 1.

COG		N° Accession in *Frankia alni* ACN14a	Gene Name	Product	Genetic or Functional Paralog *	Localization ^#^
**Several Copies**						
C	Energy production and conversion	FRAAL2393	hupL1	Uptake hydrogenase large subunit	FRAAL1829	
		FRAAL2391	hupD1	Hydrogenase maturation protein	FRAAL1828	
		FRAAL2392	hupS1	Uptake hydrogenase small subunit precursor	FRAAL1830	SP
		FRAAL3522		Putative Formyl-CoA transferase	FRAAL4675	
		FRAAL3876		Putative acyl-CoA transferases/carnitine dehydratase	FRAAL4764	
		FRAAL2565		Putative polyketide oxygenase/hydroxylase	FRAAL4792, FRAAL2325, FRAAL3051, FRAAL3395	
		FRAAL3041		Putative Dihydrolipoamide acyltransferases	FRAAL5152	
E	Amino acid transport and metabolism	FRAAL6516		Putative membrane protein	FRAAL1256	TM
I	Lipid transport and metabolism	FRAAL2505	atoD	Acetoacetyl-CoA transferase	FRAAL2504, FRAAL3148, FRAAL3149	
		FRAAL4765		Putative enoyl-CoA hydratase	FRAAL2509, FRAAL2514, FRAAL3092, FRAAL3517, FRAAL3973, FRAAL5910, FRAAL6774	
		FRAAL1660		Putative Acyl-CoA dehydrogenase	FRAAL6459	
J	Translation, ribosomal structure and biogenesis	FRAAL4260		Putative glutamyl-tRNA(Gln) amidotransferase, subunit A	FRAAL0363, FRAAL3665, FRAAL6013, FRAAL6173	
K	Transcription	FRAAL2359		Putative tetR family transcriptional regulator	FRAAL4751	
		FRAAL1892		Putative HTH-type transcriptional regulator	FRAAL4821	
		FRAAL6046		Transcriptional regulator (MerR-family)	FRAAL6751	
		FRAAL1282		Putative merR family transcriptional regulator	FRAAL6823	
L	Replication, recombination and repair	FRAAL5342		Hypothetical protein; putative DNA helicase IIhomolog	FRAAL0267	
		FRAAL6137		Putative ribosylglycoyhydrolase	FRAAL0303, FRAAL5802, FRAAL6736	
P	Inorganic ion transport and metabolism	FRAAL1452		Putative ABC transporter, permease protein	FRAAL1453, FRAAL1557	TM
Q	Secondary metabolites biosynthesis, transport and catabolism	FRAAL3901		Putative Phytoene dehydrogenase	FRAAL2168	
R	General function prediction only	FRAAL0277	surE	Acid phosphatase SurE, survival protein.	FRAAL6200	SP
T	Signal transduction mechanisms	FRAAL3898		Hypothetical protein	FRAAL6520	
	NI	FRAAL6489		Hypothetical protein	FRAAL1398	TM
		FRAAL1769		Hypothetical protein	FRAAL5611	
**Single copy**						
C	Energy production and conversion	FRAAL1457		Putative Xanthine dehydrogenase		
		FRAAL4787		Putative N-glycosyltransferase		
		FRAAL3448	glpQ	Glycerophosphoryl diester phosphodiesterase		SP
D	Cell cycle control, cell division, chromosome partitioning	FRAAL2959		ATP/GTP binding protein		TM
E	Amino acid transport and metabolism	FRAAL5354		Hypothetical protein		
		FRAAL4450		Putative Monomeric sarcosine oxidase (MSOX)		
		FRAAL1891		Putative sarcosine oxidase subunit β		
		FRAAL4839		ABC peptide transporter		SP
F	Nucleotide transport and metabolism	FRAAL3674		Uridine kinase		
G	Carbohydrate transport and metabolism	FRAAL0592		Putative ROK family transcriptional regulator		
H	Coenzyme transport and metabolism	FRAAL6157		Conserved hypothetical protein; putative Pantothenate kinase		
I	Lipid transport and metabolism	FRAAL2810		Hypothetical protein		
K	Transcription	FRAAL0335		Putative LuxR family transcriptional regulator		
		FRAAL1455		Hypothetical protein		
		FRAAL1658		Putative two-component system response regulator		
		FRAAL2338		Hypothetical protein		
		FRAAL2354		Putative DNA-binding protein		
		FRAAL3054		Hypothetical protein		
		FRAAL3611		Putative MarR family transcriptional regulator		
		FRAAL3970		Putative repressor		
		FRAAL3977		Putative TetR-family transcriptional regulator		
		FRAAL4738		Putative LuxR-family transcriptional regulator		
L	Replication, recombination and repair	FRAAL0558		Conserved hypothetical protein; putative DNA-glycosylase domain		
L	Replication, recombination and repair	FRAAL4221		Hypothetical protein		
O	Posttranslational modification, protein turnover, chaperones	FRAAL1895		Putative heat shock protein 16		
		FRAAL2394		Thioredoxin-like protein		
		FRAAL5033		Putative alkaline serine protease		SP
P	Inorganic ion transport and metabolism	FRAAL3036		Hypothetical protein		
		FRAAL3387		Cyclohexanone monooxygenase		SP
		FRAAL3502		Hypothetical protein; putative Rieske [2Fe-2S] domain		
R	General function prediction only	FRAAL0327		Putative amidohydrolase		
		FRAAL5340		Hypothetical protein		
		FRAAL3906		Putative integral membrane transport protein		TM
		FRAAL3907		Putative ABC-type uncharacterized transport system		TM
S	Function unknown	FRAAL1385		Hypothetical protein		
		FRAAL3029		Hypothetical protein		
		FRAAL1789		Hypothetical protein		TM
T	Signal transduction mechanisms	FRAAL1745		Tellurium resistance protein terE		
U	Intracellular trafficking, secretion, and vesicular transport	FRAAL4430		Putative signal peptide		SP
	NI	FRAAL0290		Hypothetical protein		
		FRAAL1186		Hypothetical protein		
		FRAAL6274		Hypothetical protein		
		FRAAL6706		Hypothetical protein		
		FRAAL3025	gvpA	Gas vesicle synthesis-like protein		
		FRAAL3026	gvpF	Gas vesicle protein F		
		FRAAL1685		Putative IMP dehydrogenase/ GMP reductase domain		
		FRAAL1686		Putative P-loop containing nucleotide triphosphate hydrolase domain		
		FRAAL2305		Hypothetical protein		
		FRAAL2306		Hypothetical protein		
		FRAAL2795		Hypothetical protein		
		FRAAL3310		Hypothetical protein		
		FRAAL3311		Hypothetical protein		
		FRAAL3894		Hypothetical protein		
		FRAAL4437		Hypothetical protein		
		FRAAL4895		Hypothetical protein		
		FRAAL4893		Putative N-acetylmuramoyl-L-alanine amidase domains		SP
		FRAAL0360		Putative signal peptide		SP
		FRAAL5030		Putative signal peptide		SP
		FRAAL5032		Putative signal peptide		SP
		FRAAL4294		Putative signal peptide		SP
		FRAAL4721		Putative signal peptide		SP
		FRAAL5515		Putative lipoprotein		SP
		FRAAL6270		Putative signal peptide		TM
		FRAAL3669		Hypothetical protein		TM

* Paralog proteins were identified by BlastP (obtaining a coverage > 50% and a percent of identity > 30%) or by KEGG (found in the same metabolic function). ^#^ Localization was performed using SignalP6 and DeepTMHMM. “SP” and “TM” for Secreted Proteins and Transmembrane Proteins, respectively.
